# Early administration of Vitamin C in patients with sepsis or septic shock in emergency departments: A multicenter, double blinded, randomized controlled trial: The C-EASIE trial protocol

**DOI:** 10.1371/journal.pone.0259699

**Published:** 2021-11-05

**Authors:** Stefanie Vandervelden, Lina Wauters, Jan Breuls, Steffen Fieuws, Philippe Vanhove, Ives Hubloue, Magali Bartiaux, Jacques Creteur, François Stifkens, Koen Monsieurs, Didier Desruelles

**Affiliations:** 1 Department of Emergency Medicine, University Hospitals Leuven, Leuven, Belgium; 2 Department of Emergency Medicine, Algemeen Ziekenhuis Turnhout, Rubensstraat, Turnhout, Belgium; 3 Leuven Biostatistics and Statistical Bioinformatics Center (L-BioStat), Kapucijnenvoer, Leuven, Belgium; 4 Department of Intensive Care, GZA Ziekenhuizen, Antwerpen, Belgium; 5 Department of Emergency Medicine, University Hospitals Brussel, Jette, Belgium; 6 Department of Emergency Medicine, University Medical Center Saint Pierre, Bruxelles, Belgium; 7 Department of Intensive Care, Erasme Hospital Brussels, Bruxelles, Belgium; 8 Department of Emergency Medicine, Center Hospitalier Universitaire de Liège, Liège, Belgium; 9 Department of Emergency Medicine, University Hospitals Antwerp, Edegem, Belgium; UNITED KINGDOM

## Abstract

**Background:**

Sepsis is a potentially life-threatening condition characterized by a deregulated body’s response to infection causing injury to its own tissues and organs. Sepsis is the primary cause of death from infection. If not recognized and treated timely, it can evolve within minutes/hours to septic shock. Sepsis is associated with an acute deficiency of Vitamin C. Despite the proof-of-concept of the benefit of administering Vitamin C in patients with sepsis or septic shock, Vitamin C administration is not yet current practice.

**Objective:**

To investigate the potential benefit of early administration of high doses of Vitamin C in addition to standard of care in patients with sepsis or septic shock.

**Methods:**

This phase 3b multi-center trial is conducted in 8 hospitals throughout Belgium. In total 300 patients will be randomly assigned to one of two groups in a 1:1 allocation ratio. The intervention group will receive 1.5 g Vitamin C 4 times a day during 4 days, started within 6 hours after admission. The primary outcome is the average post-baseline patient SOFA score.

**Conclusion:**

This trial will determine whether the early administration of Vitamin C in patients with sepsis or septic shock can lead to a more rapid solution of shock and less deterioration from sepsis to septic shock, hereby reducing morbidity and mortality as well as the length of hospital stay in this patient population.

**Trial registration:**

The C-EASIE trial has been registered on the ClinicalTrials.gov website on 10 February 2021 with registration number NCT04747795.

**Trial Sponsor:**

UZ Leuven (sponsor’s reference S63213)

## Introduction

### Background and rationale

Sepsis is a potentially life-threatening condition characterized by a deregulated body’s response to infection causing injury to its own tissues and organs. Sepsis is the primary cause of death from infection, especially if not recognized and treated timely, it can evolve within minutes/hours to septic shock [1].

Current practice guidelines for management of sepsis and septic shock are based on the Survival Sepsis Campaign (SSC) Guidelines (http://survivingsepsis.org). These international guidelines focus on early identification and treatment of sepsis by the introduction of the Hour-1 bundle. The Hour-1 bundle encourages clinicians to act as quickly as possible to obtain blood cultures, administer broad spectrum antibiotics, start appropriate fluid resuscitation, measure lactate, and begin vasopressors if clinically indicated [2].

The adoption of a ‘one-size-fits-all’ protocol has been the largest improvement in sepsis management we have seen so far. However, despite implementation of these guidelines, mortality is still high. This is reflected in the international SSC Database with over 130 000 registered patients. This database reports an overall mortality rate of 34.8% [3].

The severity of sepsis and the outcomes of sepsis and septic shock are dependent on the nature of infection and the inflammatory response it provokes. This has led to the search for targeted agents that limit the inflammatory cascade, including Vitamin C.

Vitamin C is a cofactor in the synthesis of endogenous adrenaline and cortisol, and also plays a role in mediating inflammation[4, 5]. Sepsis is associated with an acute deficiency of Vitamin C[6–8]. Despite the proof-of-concept of the benefit of administering Vitamin C in patients with sepsis or septic shock, Vitamin C administration is not yet current practice. This is mainly due to the large variety in study set-ups, primary and secondary outcomes and inconsistent results.

In 2014, Fowler et al. performed a single center Randomized Controlled Trial (RCT) involving the administration of two different doses of Vitamin C to patients with severe sepsis [9]. Vitamin C reduced the Sequential Organ Failure Assessment (SOFA) score in a seemingly dose dependent fashion.

In 2016, Zabet et al. performed a double blinded single center RCT involving the administration of IV Vitamin C to patients with vasopressor-dependent septic shock [10]. Vitamin C caused a significant reduction in the duration of vasopressor infusion and an improvement in 28-day mortality.

The before-after study by Paul Marik et al. was the first one investigating the use of Hydrocortisone, Ascorbic Acid and Thiamine (HAT) in septic shock. This resulted in a dramatic decrease in mortality (8.5% in HAT group vs 40.4% in control group) [11]. Since then several RCTs [12–19] have evaluated vitamin C alone or as part of HAT therapy in sepsis and septic shock (Table 1).

**Table 1:** Summary of RCTs evaluating Vitamin C or HAT therapy (Hydrocortisone, Ascorbic Acid and Thiamine) in sepsis and septic shock.

					RESULTS		
Study	Intervention	Sample size	Time to treatment (hrs)	Primary	ΔSOFA	Mortality	Time off vasopressors
CITRIS-ALI, 2019	A	167	?	ΔSOFA at 4 days + shock resolution	No Difference	Improved	Not reported
ACTS, 2020	HAT	200	13,5	ΔSOFA at 3days	No Difference	No Difference	No Difference
ATESS, 2020	AT	111	3,3	ΔSOFA at 3 days	No Difference	No Difference	No Difference
ORANGES, 2020	HAT	137	9,9 (3–14)	ΔSOFA at 4 days + shock resolution	No Difference	No Difference	Improved
HYVCTTSSS, 2020	HAT	80	?	28-day mortality	Improved	No Difference	No Difference
Wani et all, 2020	HAT	100	<24	In hospital mortality	No Difference	No Difference	Improved
Vitamins, 2020	HAT	216	12,1	Time alive + free of vasopressors at day 7	Improved	No Difference	No Difference
VICTAS, 2021	HAT	501	14,7	Ventilator- and vasopressor-free days	No Difference	No Difference	No Difference

Two recent meta-analyses found that both HAT therapy and the combined therapy of thiamine and Vitamin C significantly reduce the duration of vasopressor use and are associated with a reduced SOFA score on day 3. There was however insufficient data to analyse the delta SOFA scores in more detail [20, 21].

Although it is generally accepted that in sepsis timing of therapies matters, much like stroke and AMI, the majority of these studies started quite late (up to 12 to 48 hrs post ICU admission) in the disease course with the administration of Vitamin C. As Vitamin C potentially prevents the development of multi-organ dysfunction by treating microvascular dysfunction, mitochondrial injury and oxidative stress, this late administration might miss its intended effect [22–24]. Also, there may be specific patient populations for whom beneficial outcomes are more likely. Comparison of several trials suggests that the severity of critical illness matters [11, 18].

### Objectives

The C-EASIE trial focuses on the early administration of Vitamin C, within 6 hours after arrival in the ED, in patients in earlier stages of the disease course. We opted for a high dose IV Vitamin C only adjuvant therapy, in order to investigate the benefit of mono therapy. Hydrocortisone and thiamine are certainly indicated in certain patient groups but whether HAT therapy induces additional benefit remains uncertain. By setting up this pragmatic comparative effectiveness trial, we hope to see a more rapid solution of shock and less deterioration from sepsis to septic shock, hereby reducing morbidity and mortality as well as the length of hospital stay in this patient population.

## Material and methods

### Study design and setting

The C-EASIE trial is a prospective, multi-center, double blinded randomized controlled trial (RCT) in patients presenting at the ED with sepsis or septic shock that investigates the potential benefit of early administration of high doses of Vitamin C (1.5 g bolus, 4 times a day during 4 days, started within 6 hours after admission) in addition to standard care. Patients will be randomly assigned to one of two groups (physiologic serum or Vitamin C). All other aspects of care will be the same for both groups.

The study is a phase 3b multi-center RCT conducted in 8 hospitals throughout Belgium: Universitair Ziekenhuis Leuven, Universitair Ziekenhuis Antwerpen, Universitair Ziekenhuis Brussel, Algemeen Ziekenhuis Turnhout, Centre Hospitalier Universitaire de Liège, Université Libre de Bruxelles Hôpital Erasme, Centre Hospitalier Universitaire St-Pierre Bruxelles and Gasthuiszusters Antwerpen. Patients will be recruited at the EDs of the participating sites.

### Ethical approval

The study (protocol v2.0, 6/4/2021) received approval from the ethical committee and competent authorities through the CTR Pilot Procedure of the Federal Agency for Medicines and Health Products in Belgium (Pilot 389_2020-0001862-12) on 4/6/2021 and has been registered on the ClinicalTrials.gov website on February 10, 2021 with registration number NCT04747795. The trial will be conducted according to the Declaration of Helsinki.

Written informed consent (IC) will be obtained from study subjects prior to the start of study medication administration. If and only if the study subject is unable to write, oral consent in the presence of at least one witness of legal age may be given. If a study subject is unable to read, an impartial witness will be present during the entire IC process and discussion. If a study subject is unable to speak or read Dutch, French or English, an interpreter will be present during the entire IC process and discussion. When the study subject is not capable to give prior written or oral consent due to the subject’s critical condition, the study subject can be enrolled in the study on the condition that an ICF has been signed and provided by the study subject’s legally acceptable representative (LAR). When prior written or oral consent of the study subject is not possible due to the subject’s critical condition, enrollment can take place without consent if and only if the study subject is not accompanied by a LAR and the study subject’s LAR is not able to accompany the study subject or provide oral permission within 6 hours.

### Eligibility

Adult patients who present themselves at the ED with sepsis or septic shock will be checked for eligibility and included if informed consent is obtained.

Inclusion criteria:

- Patient is ≥ 18 years old.
- Patient has a ‘suspected infection’: this requires the combination of antibiotic administration and body fluid cultures within the first 6 hours after ED presentation.
- Patient has a National Early Warning Score (NEWS) ≥ 5.

NEWS is based on a simple aggregate scoring system in which a score is allocated to physiological measurements, already recorded in routine practice, when patients present to the hospital. Six simple physiological parameters form the basis of the scoring system: respiration rate, oxygen saturation, systolic blood pressure, pulse rate, level of consciousness or new confusion and temperature.

Exclusion criteria:

- Antibiotics administered as a single dose or as a prophylactic treatment.
- Antibiotics administered without an accompanying body fluid culture according to the timeframe (within 6 hours after ED presentation).
- ‘Do no intubate’ or ‘comfort measures only’ status.
- Failure to randomize within 6 hours after ED presentation.
- Weight < 45 kg.
- Pregnant or breastfeeding.
- Known allergy for Vitamin C.
- Known history of oxalate nephropathy or hyperoxaluria.
- Known history of glucose-6-phosphate dehydrogenase deficiency.
- Known history of chronic iron overload due to iron storage and other diseases.
- The patient is already on IV steroids for a reason other than septic shock.
- Proven active COVID-19 infection (positive swab and/or CT scan positive for COVID-19 within 14 days prior to or at ED presentation).
- Participation in an interventional trial with an investigational medicinal product (IMP) or device.

### Screening and consent

Patient identification will happen at the triage of the ED. Adult patients with suspected infection are flagged. The principal investigator or sub-investigator will be informed. An information leaflet and poster will be available at triage to ensure the timely identification of potentially eligible patients.

The aim is to conduct the trial on the basis of prior informed consent by the trial participants and/or their LAR. However, some of these patients are critically ill and unable to give informed consent. To ensure the timely start of medication administration (within 6 hours), a process of delayed consent, enrolling patients into the clinical trial and obtaining consent as soon as practical from either the patient or their LAR, will be applied if necessary.

The process for obtaining and documenting initial and continued informed consent from potential trial participants will be conducted in accordance with ICH-GCP E6(R2), applicable regulatory requirements and internal standard operating procedures.

Eligibility will be logged in the screening log. This will be in a strictly anonymous manner, and in no way (nor through the sponsor, nor through the investigator) this information can be traced back to the patient. This way we will generate an anonymous, aggregate data set. Screen failures will include patients who have consented but have not received treatment in a timely way or meet one of the exclusion criteria.

The patient will be logged in the subject identification log and receive a study specific subject number.

### Randomization

Patients will be randomly assigned to one of two groups in a 1:1 allocation ratio stratified by site through the digital platform Randomize.net. Block size is 4. Ardena Gent NV, a Drug Product Development & Manufacturing firm, will blind, package, relabel the IMP kits and provide randomization. Site staff will be blinded to study arm allocation.

### Intervention

Once the patient has been randomized, a patient treatment kit will be allocated. The treatment kit will contain ampoules of Vitamin C 500mg/5ml or of Normal Saline 5ml 9mg/ml. These ampoules have identical sizes and will be blinded by a cap and sticker for an identical look. Every 6 hours, bedside nurses will have to dilute 3 ampoules in 50ml of Normal Saline. In total the patient will receive 16 doses of study medication over 4 days. A maximum of 8 hours is allowed between 2 doses and only one dose can be missed. If these criteria are not fulfilled the patient will be excluded from the trial.

Treatments kits are stored locally in every ED in a temperature controlled manner (15–25°C). Once the treatment kit is assigned, the kits stays with the patient throughout his/her hospital stay.

Other than the administration of study medication and the procalcitonin testing, all management of randomly assigned patients will be at the discretion of the clinical team.

However, to standardize as much as possible the management of sepsis patients, a sepsis protocol was made in consultation with the participating sites and according to the latest evidence based standards of care in sepsis. This sepsis guideline is part of the protocol and can be used without obligation.

### Trial procedures

Parameters will be obtained as part of routine clinical care. To avoid burden on clinical care we will mainly collect lab values that are considered routine clinical care in sepsis. Only on day 1 and 4 a targeted blood sample will be collected for procalcitonin determination.

Baseline data will be obtained as close as possible to the time of randomization and will include patient demographics, NEWS at ED presentation, SOFA score at randomization, sepsis etiology, disease severity, physiologic variables, level of respiratory support, vasopressor and renal replacement therapy (RRT) use, fluid balances and time from ED presentation to the 1st dose of antibiotic and Vitamin C/placebo.

The EQ-5D-5L health questionnaire will be obtained on admission, day 5, day 28 and after 3 months. If the patient’s clinical status doesn’t allow self-completion, the questionnaire may be completed by proxy.

Arterial Blood Gas (ABG) analysis is part of routine clinical care in the Intensive Care Unit. If the patient would be discharged to the ward before day 5, ABG sampling is not considered standard of care. In order to be able to calculate the SOFA score in this population, we will use the SpO2/FiO2 ratio to impute for PaO2/FiO2 ratio in the respiratory component [25].

**Fig 1:**
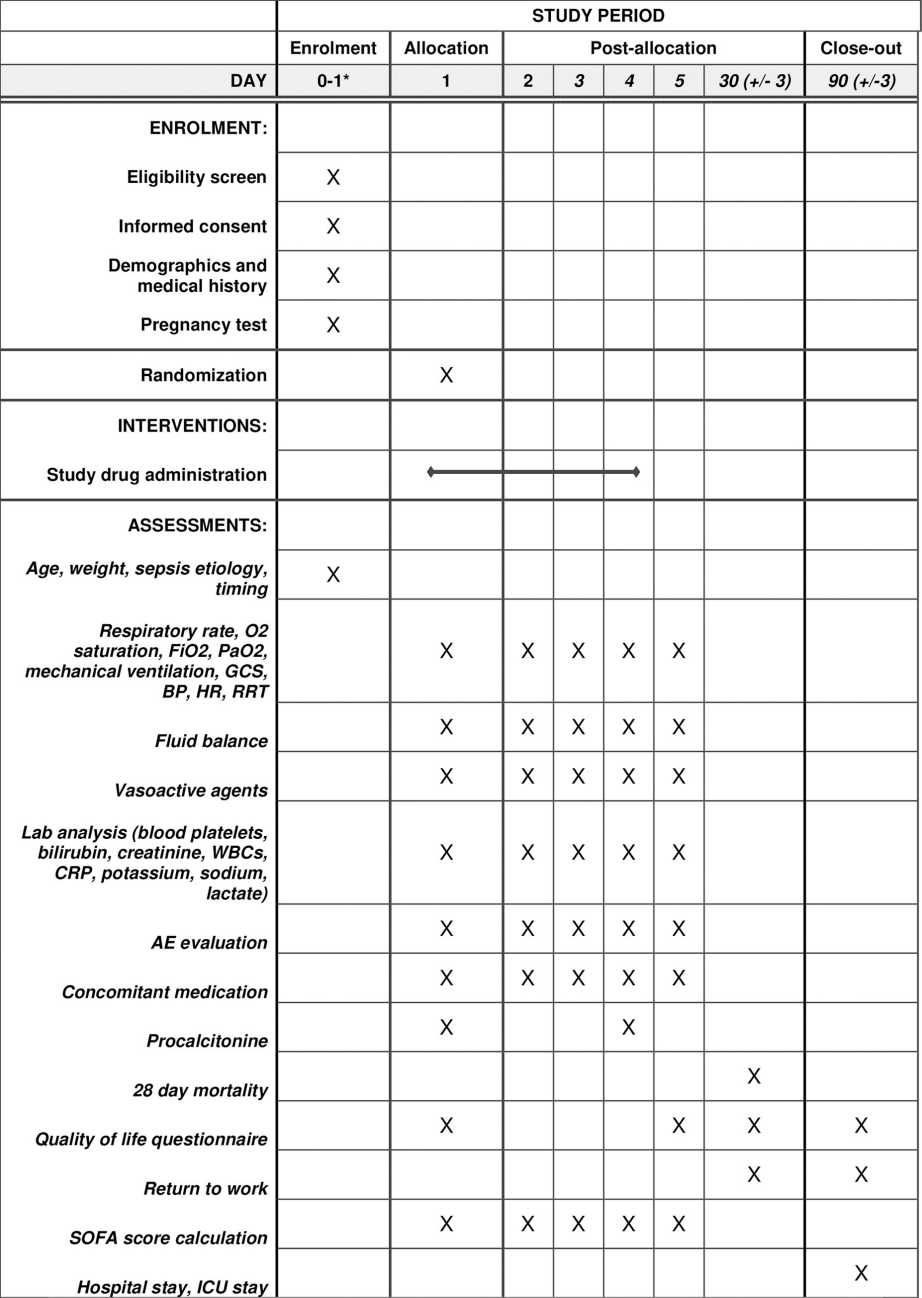
SPIRIT schedule including all trial relate procedures. FiO2: Fraction if inspired Oxygen; PaO2: Partial Pressure of Oxygen; GCS: Glasgow Coma Scale; BP: blood pressure; HR: Heart Rate; RRT: Renal Replacement Therapy; SOFA: Sequential Organ Failure Assessment; ICU: Intensive Care Unit. * Only when a patient is admitted at the ED after 6 PM, it might be that screening and randomization will not take place on the same date. In that case screening will be performed on day 0. Otherwise both screening and randomization will be performed on the same day, being day 1. ** Concomitant medication: additional information about the administration of vasoactive agents, fluids and corticosteroids will be collected from day 1 to 5. This needs to be registered in different sections of the electronic Case Report Form. The only concomitant medication that needs to be specified are antibiotics and the medication used to treat potential adverse events. Continuation of home medication, pain medication thrombosis or ulcer profylaxis are not considered as concomitant medication.

**Fig 2:**
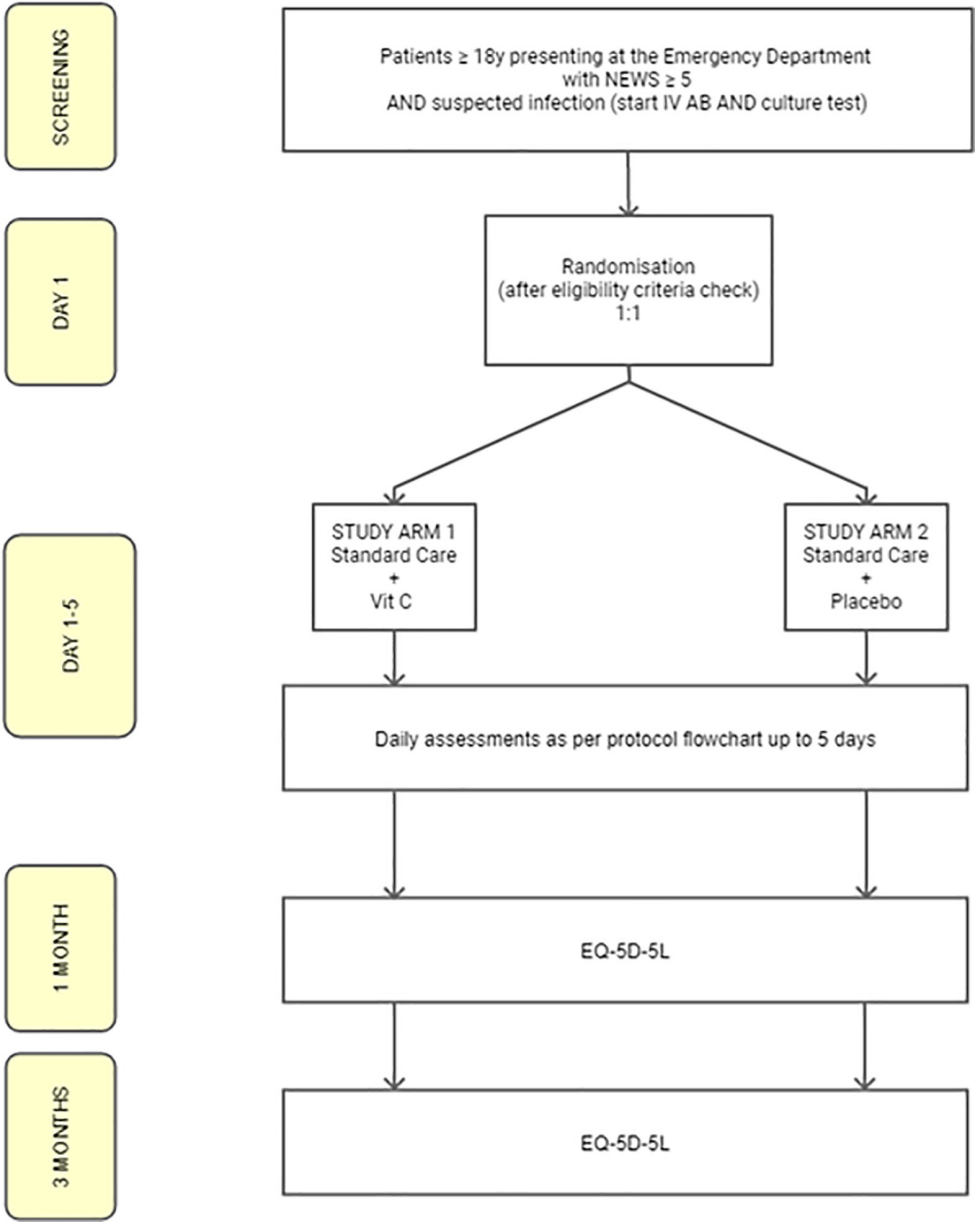
Patient Flow Chart.

All trial related procedures can be found in the SPIRIT schedule in Fig 1 and the patient flow in Fig 2.

### Outcomes

The clinical outcomes of this trial have been chosen based on core clinical outcome measures in critically ill patients and clinical outcome measures used in previous studies with Vitamin C.

#### Primary outcome

Average post-baseline patient SOFA score on day 1 to 5 after patient randomization.

To avoid missing data for deceased patients, the maximum SOFA score of 24 will be assigned to mortality. Missing values for the non-deceased patients will be imputed (see statistical analysis plan). In this model the mean SOFA score is compared between both groups at each of the post-baseline days (d2, d3, d4, d5). Hence, at each post-baseline day there will be an estimate of an intervention effect.

### Secondary outcomes

- 28-day mortality
- Maximum SOFA score
- Length of ICU and length of hospital stay
- Duration and dosage of vasopressor requirement
- RRT duration and need
- Number of ventilator days
- Total dose of steroids given
- Quality of life measured using the EQ-5D-5L questionnaire on days 1, 5, 28 and after 3 months
- Time to return to work (if applicable)

### Statistical plan

#### Sample size

The required sample size is calculated to detect with at least 80% power a difference in average post-baseline patient SOFA score (calculated over days 1 to 5), based on a constrained longitudinal data analysis model with alpha set at 0.05 [26]. The power is calculated using an approach presented by Stroup [27]. Patients who die within the time period of 5 days receive the highest SOFA score starting at the day of death. Based on the cLDA model, 126 patients per group are needed to detect with at least 80% power a difference between the control and intervention group of 1 in average post-baseline SOFA score. A standard deviation of the SOFA score equal to 3.5 and a correlation between the time points equal to 0.5 is assumed. These were conservative estimates, derived from reported information in two studies [9, 18]. 2.5%, 5%, 10% and 15% missing values not due to death are assumed on d2, d3, d4 and d5, respectively (combination of dropout and mortality). However, the sample size will be increased to 150 patients per group to anticipate a larger variability due to the imputation of one or more maximal SOFA scores for deceased patients.

#### Statistical methods

All analyses will be performed using SAS software, version 9.4 of the SAS System for Windows.

#### Summary of baseline data

Baseline continuous data will be summarized using means (SD) or medians (IQR). For categorical data, numbers and proportions will be reported (with the corresponding sample size numbers). A consort flow diagram will be produced.

#### Primary analysis

The primary analysis compares the average post-baseline patient SOFA score (average over days 1–5) instead of the SOFA score of a single time point being either a fixed day or the day the maximum SOFA score has been reached for the following reasons. First, it is important to capture the full evolution during the first 5 days instead of selecting a single time point (being a fixed day or the day of discharge). Second, patients with septic shock will already have a high SOFA score at inclusion, such that a change versus the maximum value will not contain a lot of information. The average post-baseline score will be compared between both groups using a two-sided test derived from a cLDA model with alpha equal to 0.05. The choice of the covariance structure for the five measurements in the cLDA model will be based on the Aikake criterion. Site will be added as a fixed factor in the model. The cLDA analysis is valid under the missing at random assumption and assumes that the probability that an observation is missing only depends on the observed values of the individual, but not on the missing ones.

#### Secondary analysis

To get a deeper insight in the potential effect on the SOFA score, time point specific comparisons (at d2, d3, d4 and d5) obtained with the cLDA model will be reported. Further, the maximal SOFA score will be compared using a two-sided unpaired t-test. This comparison will be based on a multiple imputation approach (20 imputed datasets) since the observed maximum can be underestimated for patients with missing SOFA scores. Values will be imputed from the cLDA model.

For the continuous secondary endpoints (e.g. daily dosage of vasopressor requirement, daily dosage of IV fluids, lab values…) mortality will not explicitly be included in the outcome definition (such as for the primary outcome). Instead, the comparison of both groups will be based directly on a multivariate longitudinal model (applying the aforementioned criteria to select the covariance structure and with site added as a fixed factor).

To appropriately evaluate differences in length of hospital stay and length of ICU stay, death during hospital (ICU) stay will be treated as a competing risk [28] using a stratified Gray’s test for the comparison of both groups. The same approach holds for the comparison of the number of ventilation days, duration of vasopressor requirement, RRT duration and total of IV fluids administered. 28-day mortality will be compared using a stratified χ^2^ test.

#### Subgroup analysis

Subgroup analyses for the primary and selected secondary outcomes will evaluate the treatment effect across the following subgroups: age groups, disease severity at baseline (sepsis vs. septic shock) and baseline SOFA (> 8 or < 8). A forest plot will display confidence intervals across subgroups. Interaction tests will be conducted to determine whether the effect of treatment varies by subgroup.

#### Safety analysis

Safety endpoints are described above. These events will be analyzed univariate and as a composite endpoint. Time-to-event methods will be used for death and the composite endpoint. Each Adverse Event (AE) will be counted once for a given participant and graded by severity and relationship to sepsis/septic shock or study intervention. AEs leading to premature discontinuation from the study intervention and serious treatment-emergent AEs will be described as part of the primary publication of the study results.

### Safety

#### Adverse event reporting

For this trial, only AEs graded Severe and Serious Adverse Events (SAE) will be collected. However, given that this patient population is often very ill, AEs expected in the disease progression of the patient or related to the standard of care for the patient will not be recorded for the study. AEs that are also outcomes of the trial, are also exempt from reporting. All AEs will be reported until 30 days after last trial treatment administration or until last follow-up visit, whichever occurs first.

Investigators will seek information on AEs during each patient contact. If someone involved in the patient’s care reports an AE that is not related to the standard of care or that is not expected in the disease progression, the site study team will evaluate the AE for seriousness, severity and causality. All AEs graded severe and SAEs will be reported to the sponsor, who will also perform a causality assessment. If a causal relationship is observed, expectedness will be evaluated and if needed SUSARs will be reported to the competent authorities.

#### Risk

In this critically ill patient population AEs are expected. However, since the safety of Vitamin C [29] has been well established over the years, we do not foresee any SUSARs.

### Data safety monitoring board

Data and safety monitoring will be conducted by an independent Data Safety Monitoring Board (DSMB) to ensure and maintain the scientific integrity and ethical balance of human subjects’ research and to protect subjects from avoidable harm. As detailed in its charter, the c-easie trial DSMB will be composed of 4 individuals: one emergency physician, one critical care physician, one statistician and one quality engineer. These individuals are selected on the basis of their content expertise in sepsis, critical care, multi-center clinical trials, and adaptive trial design and implementation. The DSMB will meet at least twice a year until study completion and will report to the c-easie executive committee. The DSMB will act independently of the funder and sponsor of the study and is charged with ensuring that the trial is implemented as designed and that the pre-specified design continues to be scientifically and ethically appropriate, and the DSMB will review ongoing safety data.

### Trial steering committee

The role of the Trial Steering Committee (TSC) is to provide the overall supervision of the trial. The TSC will also include members who are independent of the investigators, their employing organisations, funders and sponsors. The TSC should monitor trial progress, conduct and advise on scientific credibility. The TSC will consider and act, as appropriate, and ultimately carries the responsibility for deciding whether a trial needs to be stopped on grounds of safety or efficacy.

The TSC will meet on average 3 times per year the first year and twice the year after that. The TSC is composed of the CI, the trial statistician, the trial PM, an independent expert, a representative of other participating centres or groups, 2 patients or members of the public, 1 representative of the sponsor and 1 representative of the funder.

The day-to-day management of the study will be performed by the Trial Management Group (TMG) which is distinct from the TSC.

### Data management

Source records in this trial are the electronic patient dossier and paper EQ-5D-5L questionnaires. All study data are collected by trained staff at each study site. Data collection includes baseline demographics, primary diagnoses, physiological parameters and pathology, interventions and documentation of outcomes and safety events. Data are entered in the electronic Case Report Form (eCRF) on the secure web platform REDCap. All participant data will be pseudonymized using a unique study-specific identifier for each trial participant, in compliance with applicable data protection regulations. No personal data will be collected in the eCRF. Only study team members, monitors and auditors/inspectors for whom the chief investigator has requested project-specific eCRF access, are granted access to the clinical database. Upon successful training completion each user is centrally assigned a user role, associated with predefined system/data privileges. Following periodic data reviews, the data will be cleaned using an interactive query workflow whereby the data manager and/or safety reviewer and/or monitor will open a query when identifying missing and/or discrepant and/or unsubstantiated data, prompting the investigator and/or designated study team members to address the issue. Final study data will be archived electronically on UZ servers and with access rights for KCE and any other Belgian federal or regional institution, body, office, public service and/or agency at the end of the Study.

### Monitoring

Data monitoring will be conducted by the CTC of UZ Leuven. The Site Initiation Visit is conducted on each site after contractual agreements have been duly signed. As detailed in the monitoring plan, a first monitoring visit will be conducted within 6 weeks after inclusion of the first study subject at the site. Thereafter, monitoring visits will be organized at intervals of 4 months on average. In total 25% of the total number of expected study subjects will be monitored, and 100% source data verification will be performed for the first 2 study subjects at each site. The remaining subjects will be randomly selected for review of critical data. A close-out visit will be conducted after the last patient last follow-up questionnaire has been completed for the site.

### Interim analysis

Given the uncertainty on the assumed variability of the modified SOFA score and on the assumed correlation between the time points, a blinded interim analysis for sample size re-estimation [30] will be performed 12 months after start of the study (or at the latest if 75% of the planned number of subjects is recruited). If the observed standard deviation and correlations deviate from the assumed values such that the desired power level of 80% is not guaranteed anymore, an increase of the planned sample size will be considered. If the power level is at least 80% with the observed values for the CV, the sample size will remain unaltered. Note that no interim analyses are planned to stop the study earlier for efficacy or futility, this to avoid loss of information on the secondary endpoints.

### Timeline

Sites gradually started patient recruitment from June 2021. The Trial will run for 18 months. At the end of the inclusions three months of patient follow-up will be added. We expect to submit for publication in the autumn of 2023.

### Dissemination policy

The Declaration of Helsinki (latest version) and European and Belgian regulations require that every research Trial involving human participants be registered in a publicly accessible database before recruitment of the first participant. The CI is responsible for registering the Trial.

In addition, the CI will fulfil their ethical obligation to disseminate and make the research results publicly available. As such the CI is accountable for the timelines, completeness and accuracy of the reports. Researchers, authors, Sponsors, editors and publishers must adhere to accepted guidelines for ethical reporting. Negative and inconclusive, as well as positive results must be published or otherwise made publicly available. Sources of funding, institutional affiliations and conflicts of interest must be declared in publication.

### Authorship eligibility guidelines and any intended use of professional writers

It is anticipated that the results of the overall Study shall be published in a multi-center publication, involving the data of all clinical sites participating in the Study.

A Participating Site is not allowed to publish any data or results from the Study prior to the multicenter publication, provided however that Participating Site is allowed to publish the results generated at the Participating Site if the multicenter publication has not occurred after 12 months from Study database lock.

Any publication by Participating Site will be submitted to the Sponsor for review at least thirty (30) days prior to submission or disclosure. Sponsor shall have the right to delay the projected publication for a period of up to three (3) months from the date of first submission to the Sponsor in order to enable the Sponsor to take steps to protect its intellectual property rights and know-how.

Publications will be coordinated by the Investigator of Sponsor. Authorship to publications will be determined in accordance with the requirements published by the International Committee of Medical Journal Editors and in accordance with the requirements of the respective medical journal.

## Discussion

Recently, numerous RCTs have been published (Table 1). Moreover, at the time of this writing, nine RCTs on the use of Vitamin C in sepsis (in addition to the c-easie trial), are registered at clinical trials.gov (accessed 7-9-2021) and have the status planned, recruiting or not yet published. Of these studies, two compare vitamin C with placebo, one compares the combination of vitamin C and thiamine with placebo (NCT03680274 and NCT03829683), two compare vitamin C and hydrocortisone with placebo (NCT03592277, NCT04999137), one compares the three-drug regimen with hydrocortisone alone (NCT03540628) and two compare the three-drug regimen with matching placebo (NCT03380507, NCT03872011).

A unique aspect of the c-easie trial compared to other registered or published trials is the early administration of Vitamin C within 6 hours after arrival at the ED. It is also the first time that the administration of Vitamin C will be studied in a sepsis population including the less ill. To detect this population more accurately, we introduced the NEWS score as a screening and inclusion tool for this trial. The NEWS score is validated for the identification of acutely ill patients in the early stage of their illness (including sepsis) being at risk for deterioration [31, 32]. This way the benefit of Vitamin C in different sepsis subgroups can be analyzed.

We aim to proof that through the early administration of Vitamin C, patients who present at an early stage of the disease course will get less sick, resulting in a lower maximum SOFA score. However, administration of Vitamin C in our sickest population might not necessarily reflect in a lower maximum SOFA score, but rather in faster recovery (higher delta SOFA between 2 points in time). To capture both phenomena, we opted to look at the average post-baseline patient SOFA score as primary outcome supported by a maximum SOFA score as secondary outcome (see statistical plan).

Furthermore we use a one drug regimen, which allows us to characterize which drug in the ‘well known’ sepsis cocktail might be paramount.

In the c-easie trial open-label steroids are permitted. As a result, patients will not be prevented by protocol from receiving stress-dose steroids if their clinical team feels this treatment is appropriate. We will consider the vasoactive effects of hydrocortisone in trial design and will assess the impact of this combination on shock resolution in comparison to placebo.

There are some limitations to this study, many of which are the result of limited phase II data. First, sample size calculations were based on only two previously published trials [9, 18]. Although conservative estimates were made, we decided to insert interim analyses for sample size re-estimation. Secondly, the large variety in study set-ups, outcomes and the inconsistency in the reported results, make it difficult to estimate the optimal dose and timing of Vitamin C administration. Based on clinical experience, ascorbic acid 1.5 grams IV every 6 hours is currently used in most studies [33]. Thirdly [4–6], since this is a pragmatic trial, we do not measure the baseline levels of Vitamin C in patients. Although several studies have examined the Vitamin C levels in sepsis patients and reported very low levels, we thus do not know with certainty whether these patients actually have a deficiency in Vitamin C. Finally, the combined population of very ill and less ill patients might influence the interpretation of the study results. It might be that Vitamin C has no benefit in the less ill population, or just the opposite. For this reason, we will perform thorough analyses of subgroups based on the NEWS score of the patients.

Any modification to the protocol which may have an impact on the conduct of the study, potential benefit of the patient or which may affect patient safety, including changes of study objectives, study design, patient population, sample sizes, study procedures or significant administrative aspects, will require a formal amendment of the protocol. Such amendment will be agreed upon by the Sponsor and KCE, and will need to be approved by the ethical committee and competent authorities through the CTR Pilot Procedure of the Federal Agency for Medicines and Health Products. If necessary, the trial will be paused while waiting for approval.

## Supporting information

S1 Checklist SPIRIT checklist. (DOC)

S1 File Data management protocol. (PDF)
