# Supplementary material for: Early administration of Vitamin C in patients with sepsis or septic shock in emergency departments: A multicenter, double blinded, randomized controlled trial: The C-EASIE trial protocol
Source: PLoS One. 2021 Nov 5;16(11):e0259699. doi: 10.1371/journal.pone.0259699 (PMC8570477; doi:10.1371/journal.pone.0259699)
Supplement: S1 File — (PDF) [file pone.0259699.s002.pdf]

GENERAL INFORMATION	
S-number & study acronym:	S63213 – KCE C-EASIE
Protocol version & date:	V 2.0 – 06 April 2021
Sponsor:	University Hospitals Leuven (UZ Leuven)
Number of research sites:	8
Expected number of participants:	300
Coordinating/Principle Investigator:	Dr. Didier Desruelles
DMP prepared/revised by:	Hilde De Tollenaere – CTC Data Manager
Study Statistician:	Steffen Fieuws
Study Safety Coordinator/Reviewer:	Dr. Stefanie Vandervelden – Johanna Geerinck CTC

Table of Contents

1. Purpose 1
2. Scope 1
3. Scope of data management activities 2
A. DATA COLLECTION 2
B. DATA ACCESS AND SECURITY 3
C. DATA STANDARDS & CODING 3
D. DATA CLEANING AND VALIDATION 3
E. RANDOMISATION / TREATMENT ALLOCATION 5
F. DATA INTEGRITY 6
G. SAFETY REVIEW / REPORTING 6
H. DATABASE LOCK 6
I. DATA RETENTION, CONTINGENCY & DISASTER RECOVERY 7
J. END OF TRIAL DATA ARCHIVING 7
K. THIRD PARTY DATA HANDLERS 7
L. INDEPENDENT DATA SAFETY MONITORING BOARD (DSMB) 8
4. Archiving 8
5. Version history 8
6. Approvals 9
7. APPENDIX 2: Data Safety Monitoring Board (DSMB) Charter 9

1. Purpose

This Data Management Plan serves to describe all study-specific clinical trial-related data management tasks and deliverables. This includes how the data are collected, how data quality and integrity is assured, how data is handled, transformed and processed, etc.

2. Scope

This DMP was developed for clinical trials for which KUL-UZ Leuven is Sponsor and/or for which data management tasks are contracted to KUL-UZL, and is governed by CTC DM-SOP-001

Out of scope:

Development and content of the Statistical Analysis Plan (SAP) and Monitoring Plan (MP)

3. Scope of data management activities

A. DATA COLLECTION				
A.1 What data will be collected or re-used and where does it have its origin?<br>The below list provides an overview of eCRF instruments. Generally spoken all data for this study has its origin in (electronic) patient file/source data worksheets. For further details about which data will be collected, please refer to the study-specific User Requirements document.				
**Data point**	**Collect**	**Re-use**	**Recording**	**Comments**
Screening	☒	☐	Source data	
Baseline data	☒	☐	Source data	
Daily follow-up	☒	☐	Source data	
Questionnaire follow-up	☒	☐	Source data or electronic patient file	
Randomisation	☒	☐	To ensure the integrity of the trial, a randomization procedure through a computerized system has been established:<br>The randomization kit list will be generated by ARDENA Gent NV and uploaded in randomize.net. The user will log into randomize.net to randomize the patient. When the patient is randomized the user will receive a kit number via mail. This kit-number will be manually entered in the eCRF by the study team.	
Health questionnaire (survey)	☒	☐	Template questionnaire (paper) can be used in case no computer is available and data can also be entered directly in the eCRF by the patient or member of the study team with data entry right	
Adverse Event	☒	☐	Source data	
Concomitant Therapy	☒	☐	Source data	
End of Trial	☒	☐	Source data	
A.2 Was UZL GDPR questionnaire completed?		☒ Yes<br>☐ No >>> *Please complete & submit to* ctc@uzleuven.be !		
A.3 Expected recruitment start date		Expected start date: 21-05-2021.<br>The study is expected to last 28 months.		

A.4 Name and version of (e)CRF platform or relational database used to capture study-specific data	REDCap™ Production version 10.6.13
A.5 Party responsible for (e)CRF development	Hilde De Tollenaere – CTC Data Manager<br>CTC.datamanagement@uzleuven.be
**B. DATA ACCESS AND SECURITY**	
B.1 Physical location of CRF database	UZ Leuven REDCap is hosted on dedicated KU Leuven data servers at Campus Heverlee.
B.2 System Administrator	For UZL REDCap the System Administrator is Gert Goos: gert.goos@kuleuven.be
B.3 How will physical data access be restricted?	When using UZ Leuven REDCap, physical access to the data centers is logged and restricted to authorized KU Leuven Information Technology (IT) personnel, using badge identification. At the clinical database level only study team members, monitors and auditors/inspectors for whom the Coordinating or Principal Investigator (as applicable) has requested project-specific eCRF access, are granted data access. Upon successful training completion each user is centrally assigned a user role, associated with predefined system/data privileges, in accordance with CTC DM-WI-001. The gatekeeper for UZL REDCap user accounts is UZL CTC (ctc.datamanagement@uzleuven.be).
B.4 Will data be shared outside UZL during and/or following completion of the clinical research trial?	☐ Yes<br>☒ No*
*If "No": Please clarify why no data will/can be shared: *This is specified in sections 11 and 12 of the protocol.*	
B.5 Describe the use and format of required data exports	For the purpose of statistical analysis and results reporting, data exports out of REDCap will be exported in SAS format.
**C. DATA STANDARDS & CODING**	
C.1 Which medical coding dictionary/dictionaries will be used?<br>*Note that safety event coding based on the MedDRA dictionary, is required for reporting study results in EudraCT.*	MedDRA,
C.2 What measures will be taken to prevent collection and sharing of personal data from trial participants?	All participant data will be pseudonymized using a unique study-specific identifier for each trial participant, in compliance with applicable data protection regulations. No personal data will be collected in the eCRF. This will be verified as part of the eCRF testing and validation.
**D. DATA CLEANING AND VALIDATION**	
D.1 Describe the type, level and frequency of quality control (QC) activities.	Data quality will be checked through reviews of comprehensive data discrepancy reports, including information about missing and unreviewed / unvalidated data fields.<br>No queries will be raised for self-evident discrepancies that appear during the process of data cleaning. These self-

	evident discrepancies will be discussed with the study team and statistician prior to being corrected by the CTC Data Manager.<br>Any missing data fields that are confirmed as missing by the study site will be considered as not available. No new queries will be raised for these missing data fields.<br>Corrections/clarifications will be asked to the data entry staff for all other discrepancies.<br>Extensive consistency checks on the received data will be done before each interim analysis and at the time of annual safety reporting.<br>The CTC Data Manager will review and close queries that have been appropriately addressed. At the end of the trial, the CTC Data Manager or the investigator will lock the eCRF page for further editing when all data for the page is complete and clean.<br>Furthermore, data integrity will also be monitored according to the agreed study-specific Monitoring Plan.
D.2 Will the study be monitored by a qualified, trained individual, who is independent from the study team?	☒ Yes<br>☐ No
If "Yes": Either describe the monitoring strategy and frequency, or refer to study-specific Monitoring Plan.<br>*If "No": Provide justification (based on documented risk analysis!) for waiving monitoring responsibilities.*<br><br>See Monitoring for this study will be done by UZL Clinical Trial Center (CTC). All UZL CTC Monitors are appropriately trained and qualified. For details about the monitoring strategy, please refer to the agreed Monitoring Plan.	
D.3 Name of monitoring party	Linde Besard<br>linde.besard@uzleuven.be<br>ctc.monitoring@uzleuven.be
D.4 Data cleaning strategy, i.e. query process	Following periodic data reviews, the data will be cleaned using an interactive query workflow whereby the Data Manager and/or Safety Reviewer and/or Monitor will open a query when identifying missing and/or discrepant and/or unsubstantiated data, prompting the Investigator and/or designated study team members to address the issue. Upon verification of response/actions taken by the study team, queries will be closed by the data manager, safety reviewer, or Monitor.<br>Several automated queries have been predefined or programmed for this study and data field validation is build-into the eCRF.
D.5 Describe how protocol deviations and/or violations will be documented and/or reported.<br>***Note**: a description of protocol deviations/ violations will be handled as part of the statistical analysis, must described as part of the SAP.*	The Investigator and Trial team acknowledge and agree that prospective, planned deviations or waivers to the protocol are not permitted under applicable regulations on clinical studies. However, should there be an accidental protocol deviation, such deviation shall be adequately documented in the source documents and on the relevant forms and reported to the CI and Sponsor immediately. Deviations should also be reported to the EC as part of the EC's continued review of the Trial (e.g. through the ASR, APR, etc.).

	Protocol deviations which are found to frequently recur, will require (immediate) action. Protocol violations will also require a documented "Corrective Action Preventive Action (CAPA)" plan.<br>Investigator acknowledges that such recurring protocol deviations could potentially be classified as a serious violation. It is understood that "a serious violation" is likely to affect to a significant degree:<br>- the safety or physical or mental integrity of the Trial participants; or<br>- the scientific validity of the Trial<br>The Investigator is expected to take immediate action required to protect the safety of the trial participant(s), even if this action represents a deviation from the protocol.<br>In such cases, the CI/Sponsor should be notified of this action and the EC at the Trial site should be informed according to local procedures and applicable regulations.<br>The impact of deviations/violations and/or missing data on the study results will be assessed by the PI and statistician. Handling of such deviations and/or data gaps will be described in the Statistical Analysis Plan (SAP) by the statistician. The statistician will alert the PI/study team/CTC Data Manager/Monitor (as appropriate), of any non-compliance issues that significantly impact the data integrity and/or validity of the study data (e.g. issues that introduce potential bias or that compromise the study endpoints).

E. RANDOMISATION / TREATMENT ALLOCATION

E.1 Is the study randomized?	☒ Yes<br>☐ No

If "Yes", please describe the randomization / treatment allocation methodology and any tools used to establish randomization / treatment allocation; or refer to the final, approved protocol if detailed information is available there.

The trail randomization is described in section 7.3 of the protocol.

To ensure the integrity of the trial, randomization through a computerized system has been established. A randomization kit list will be generated by **ARDENA Gent NV** and uploaded into www.randomize.net. Randomization will happen in a 1:1 allocation ratio stratified by site. Patients in the trial will be randomly assigned to the intervention group or the control group. Within each site, block randomization will be applied using varying block size The total number of blocks is 90, and the number of treatment numbers is between 001- 360.

www.randomize.net is a web based system and accessible 24/7. Each applicable study site staff member will have a personal log in for randomization.
When the patient has been randomized in www.randomize.net, a patient treatment kit number will be allocated. The applicable study site staff members will receive this kit number via mail.

Blinding, packaging and distribution of IMP is described in section 7.4 of the protocol:

These pre-made kits will contain the blinded IV ampoules for the entire treatment period (16 doses - 4 days) for the patient.
The treatment kit will contain 52 (4 back-up) ampoules of Vitamin C 500mg/5ml OR 52 ampoules of Normal Saline 5ml 9mg/ml. These ampoules have identical sizes and will be blinded by a cap and sticker so that they look identical. For administration, bedside nurses will have to dilute 3 ampoules in 50ml of Normal

Saline. Vitamin C for IV use and normal saline are both colorless and odorless. It has been described that Vitamin C can turn yellowish, but only if stored for too long or under non ideal circumstances.<br>This way, trial participants as well as care providers will be blinded to intervention.<br>Blinding, packaging, and distribution will also be performed by the external service Ardena Gent NV.	
**F. DATA INTEGRITY**	
F.1 How will the integrity of the data be assured during data transfer and processing?	No data imports are expected.<br>No patient identifying information is recorded in the eCRF.<br>Exports will be used as described above for data verification/queries and statistical analysis. Data will be electronically saved and are accessible only to authorized personnel. No transfers outside UZ Leuven/KU Leuven are foreseen. Transfer of data to the study statistician will be tested prior to transferring any study data.
F.2 Which measures are taken to allow verification of data integrity throughout the entire data lifecycle?	A comprehensive audit trail is maintained within the eCRF allowing to demonstrate the validity of collected trial data. This includes historical records of original data entries, by whom the data was entered and when it was entered, as well as detailed records of who, when, which and why corrections to the original data entry were made. This also includes records pertaining to managing user access and data privileges.
F.3 What measures will be taken to assure the integrity of blinded treatment allocation/information?	Not Applicable. IMP kits are blinded & labeled by Ardena Gent N.V. The site enters the IMP kit number into the eCRF.
F.4 What measures will be taken to avoid bias of independent raters? (as applicable)	Not applicable
**G. SAFETY REVIEW / REPORTING**	
G.1 How will study participant safety be assured?	The CI/PI, Safety reviewer and the monitor are notified via automated email whenever an SAE is recorded in the eCRF.<br>In section 9 of the protocol the 'Safety Recording and reporting' is described, containing the following sections:<br>9.1. Definitions<br>9.2. Recording of safety findings in function of the available evidence<br>9.3. Expedited reporting of AE's, SAEs and SUSARs<br>9.4. Reporting requirements to EC's and CA's
G.2 Party responsible for safety reviews	Dr. Stefanie Vandervelden<br>Dr. Didier Desruelles
G.3 Party responsible for safety reporting, per applicable regulations, protocol and study-specific agreements	▪ Johanna Geerinck: SUSAR reporting in Eudravigilance and to EC, CA and other investigators johanna.geerinck@uzleuven.be<br>▪ Dr. Didier Desruelles (Sponsor): Evaluation of expectedness and seriousness of the reported event didier.desruelles@uzleuven.be<br>▪ Dr. Stefanie Vandervelden: Evaluation of expectedness and seriousness of the reported events, in absence of stefanie.vandervelden@uzleuven.be<br>Lina Wauters: Reporting of SAEs and severe AEs (not SUSAR) according to the detailed guidance described in the safety SOP. lina.wauters@uzleuven.be
**H. DATABASE LOCK**	

H.1 Will an interim database lock be executed?	☐ Yes, expected date/timing for interim DB lock: ddMmmyyyy or Month/Year<br>☒ No
If "Yes", please describe timing, reason and conditions for interim database lock.	
H.2 When and under which conditions/at what point in time will the final database lock be executed?	After last follow-up visit of last patient and final data cleaning.
H.3 Expected data/timing for final DB lock	October 2023
H.4 Party responsible for interim/final database lock	Hilde De Tollenaere<br>Hilde.detollenaere@uzleuven.be<br>Ctc.datamanagement@uzleuven.be
**I. DATA RETENTION, CONTINGENCY & DISASTER RECOVERY**	
I.1 Describe contingency procedures and data backup schedule	In the UZL REDCap database, data is backed up as follows:<br>▪ The web server backup regime is specified below:<br>- An hourly backup, the last 6 versions of which are saved<br>- A daily backup, the last 7 versions of which are saved<br>- A weekly backup, the last 6 versions of which are saved<br>▪ The database backup regime is specified below:<br>- A nightly cold backup of all databases<br>- One month's storage of the nightly cold backups<br>▪ Data restore, upon request
I.2 Provide reference to relevant system disaster recovery procedures	For the UZL REDCap database, the following KU Leuven procedures for system recovery apply:<br>▪ Systems are proactively monitored 24 hours a day, 7 days a week.<br>▪ An emergency on-call service guarantees constant monitoring of the technical equipment, also outside office hours, but not at night. The on-call service is notified automatically in case of problems (between 7.00 - 23.00 hrs).<br>▪ There are no fixed maintenance windows: a timely email is sent to inform the local IT Administrator of any planned maintenance or upgrades.<br>▪ Any service unavailability, scheduled or unscheduled, is announced on the ICTS status page.<br>▪ The web space is designed redundantly: in the event of system problems on one back-end server, all traffic is automatically diverted to another back-end server. The database platform is also designed redundantly.
**J. END OF TRIAL DATA ARCHIVING**	
J.1 Describe how (format and media) data will be archived at the end of the study	Electronic format: Final study data will be archived electronically on UZ servers. In addition, each site will be supplied with their site-specific final study data set on USB-device for long-term archiving as part of the Investigator Site File (ISF)

J.2 How long will data be the study database be archived?	25 years or longer, as required per applicable regulations
J.3 Please provide archiving location following the end of the study	UZL server
**K. THIRD PARTY DATA HANDLERS**	
K.1 Are any third parties involved with any aspects of data management?	☒ Yes<br>☐ No

If "Yes", please provide name and contact details of each party and indicate whether Confidentiality Agreements (CDAs) and/or Data Transfer Agreements have been established, as appropriate:

3rd Party name	Contact details	CDA	DTA	Comments
ARDENA Gent NV	Katy Dewitte<br>Katy.dewitte@ardena.com<br>Annelies Paridaens<br>Annelies.paridaens@ardena.com<br>Lieven Van Vooren<br>LievenVanVooren@ardena.com	☐	☐	Quality Agreement (QA)<br>Technical Quality Agreement (TQA)<br>Services Agreement

**L. INDEPENDENT DATA SAFETY MONITORING BOARD (DSMB)**	
L.1 Will a DSMB be used?	☒ Yes<br>☐ No

If "Yes", please include DSMB project charter in DMP Appendix, or refer to the final, approved protocol if detailed information about the DSMB composition (members), organization (e.g. voting policy, requirements for meeting quorum, etc.), deliverables, scope, objectives and timing of DSMB activities is available in the protocol.

L.2 Frequency of DSMB meetings?	The DMC will meet prior to the start of the trial to approve the charter and protocol. After 50 patients have completed treatment, the DSMB will meet and will review the study data and advise on the continuation of the trial. They will monitor all participating sites until all patients are recruited. The DMC will then meet annually to discuss the progress of the trial.
L.3 Scope and objectives of DSMB activities?	Review the safety of the trial, described in the DSMB Charter V3.0

4. Archiving

Final versions of this DMP will be filed in the appropriate section of the study-specific TMF.

5. Version history

Version	Reason for change
1.0_22Jun2021	New document
1.1 29Jun2021	Corrections made for:<br>- Protocol version in general information<br>- Expected recruitment start date<br>- Version number DSMB charter<br>- Randomization block size<br>- Data Cleaning

Approvals

Author	CTC Head
<Signature><br>05. 08. 2021.<br><br>Hilde De Tollenaere, CTC Data Manager<br>Date:	<Signature><br><br><br>Heidi Sterckx<br>Date:
**Statistician**	**Principal Investigator**
<Signature><br><br>Steffen Fieuws<br>Date: 10/8/2021	<Signature><br><br>Dr. Didier Desruelles<br>Date: 31/08/2021

1. APPENDIX 2: Data Safety Monitoring Board (DSMB) Charter

<S63213 DSMB Charter V3.0>
